# Congenital erythropoietic porphyria (Günther disease): A rare case of early-onset photosensitivity and erythrodontia in an infant

**DOI:** 10.1016/j.jdcr.2026.06.065

**Published:** 2026-07-07

**Authors:** Rupesh Sharma, Shivam Garg, Kalindi Sud, Harsha Vardhan

**Affiliations:** Department of Dermatology, Venereology, and Leprosy, School of Medical Sciences & Research, Sharda University, Greater Noida, Uttar Pradesh, India

**Keywords:** congenital erythropoietic porphyria, erythrodontia, Günther’s disease, photosensitivity, porphyria, porphyrinuria

## Introduction

Congenital Erythropoietic Porphyria (CEP), also known as Günther disease, first described by Günther in 1911, is an ultra-rare form of porphyria with an estimated prevalence of approximately 1 in 1,000,000 live births.^1^ It is an autosomal recessive disorder caused by loss-of-function mutations in the Uroporphyrinogen III synthase (*UROS*) gene, which encodes uroporphyrinogen III synthase. This enzymatic defect leads to overproduction and accumulation of nonphysiologic type I porphyrins (uroporphyrin I and coproporphyrin I) in the bone marrow, red blood cells, and other tissues.^2^

The clinical presentation is variable, with severe forms beginning in early infancy and often leading to blistering, scarring, and mutilation of sun-exposed areas. Extracutaneous manifestations include hemolytic anemia, erythrodontia (red fluorescence of teeth), and porphyrinuria.^3^ We present a classic case of CEP in a 1-year-old male child diagnosed based on clinical features and bedside fluorescence findings in a resource-limited setting.

## Case report

A 1-year-old male child, born to a nonconsanguineous marriage, presented to the dermatology outpatient department with a history of recurrent fluid-filled lesions over the face and extremities since 4 months of age. The mother reported that these lesions appeared predominantly on sun-exposed sites, including the cheeks and dorsum of the hands and feet, and worsened following sun exposure. The blisters ruptured spontaneously within a few days and healed with crusting and hypopigmented scarring, with no history of generalized skin fragility.

The caregiver also reported red discoloration of urine that was first recognized at approximately 5 to 6 months of age and had progressively become persistent by the time of presentation, without any pain or crying during micturition (Fig 1). There was no history of similar discoloration noted at birth or during the initial episodes of blistering. There was no history of fever or drug intake prior to the onset of symptoms or any similar complaints among family members. Antenatal history was unremarkable, with no reported maternal illness or pregnancy-related complications. The child was born at term, with no history of birth asphyxia or neonatal complications.

**Fig 1:**
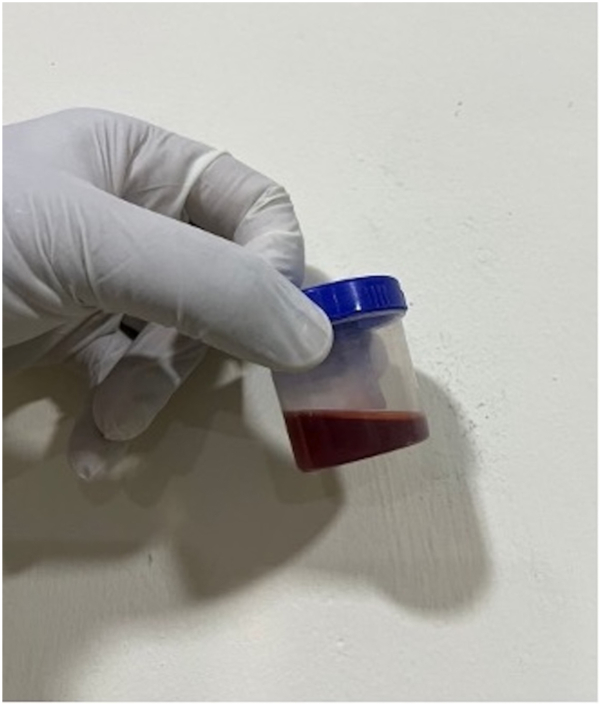
Congenital erythropoietic porphyria showing *dark red*-colored urine (porphyrinuria), a characteristic clinical finding resulting from excessive urinary excretion of porphyrins.

### Clinical examination

General examination revealed a playful child with mild pallor. Cutaneous examination showed multiple ill-defined hypopigmented patches with overlying crusting and a few atrophic scars over the face, dorsal surfaces of both hands and feet, and other sun-exposed areas. Marked facial hypertrichosis was noted, particularly over the forehead and cheeks (Fig 2, *A*-*D*).

**Fig 2:**
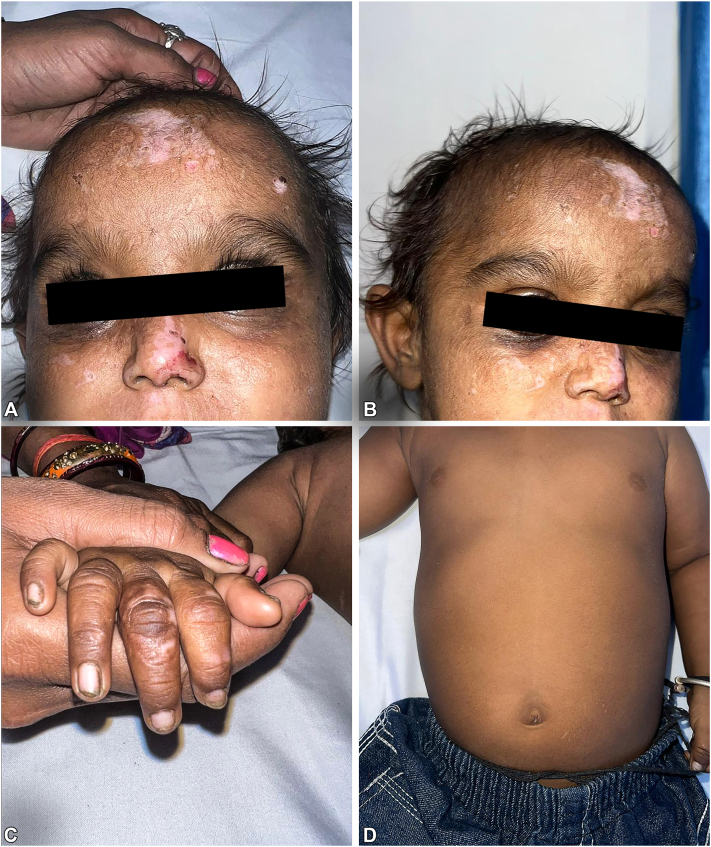
Congenital erythropoietic porphyria showing marked facial hypertrichosis **(A)** with atrophic scarring over photo-exposed areas **(A-C)** and relative sparing of photoprotected trunk skin **(D)**.

Intraoral examination revealed brownish discoloration of the deciduous teeth consistent with erythrodontia. There was no evidence of sclerodactyly or digital mutilation at presentation. Systemic examination revealed mild splenomegaly, with the spleen palpable 2 cm below the left costal margin.

## Investigations

*Wood's lamp examination*: The teeth exhibited brilliant pinkish-red fluorescence (Fig 3), and the urine sample showed bright pink-red fluorescence (Fig 4), which is highly suggestive of the presence of porphyrins.

**Fig 3:**
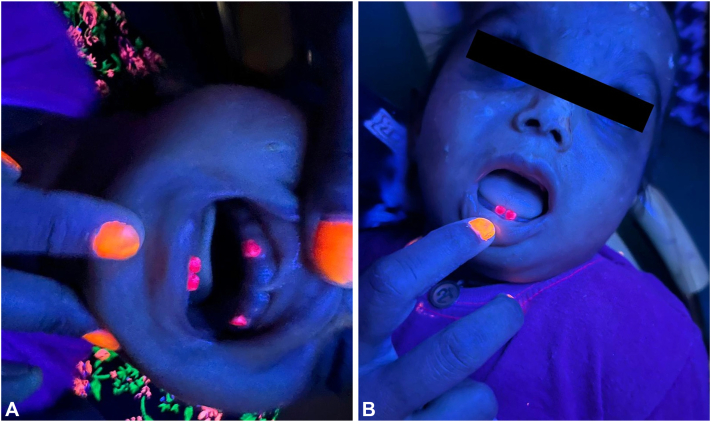
Congenital erythropoietic porphyria showing characteristic erythrodontia with pinkish-red fluorescence of deciduous teeth under Wood's lamp examination: **(A)** A close-up view of the fluorescent teeth and **(B)** a broader clinical representation of the finding.

**Fig 4:**
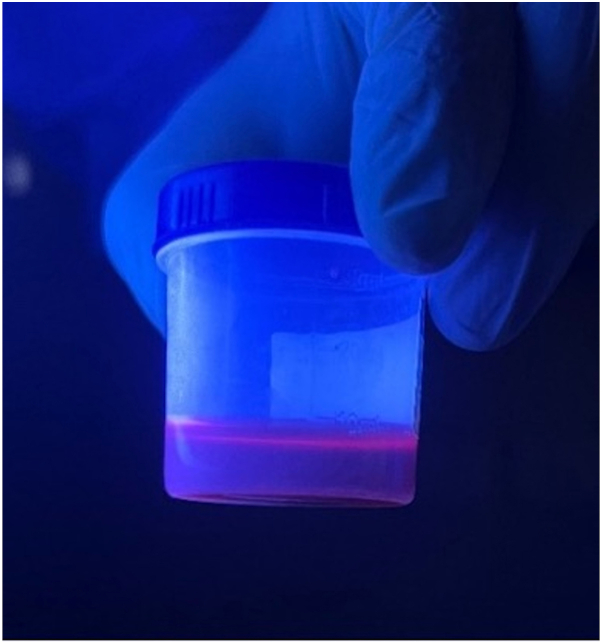
Congenital erythropoietic porphyria showing *pink-red* fluorescence of urine under Wood’s lamp due to porphyrinuria.

*Hemogram*: Hemoglobin level was 6.9 g/dL, indicating severe anemia. Peripheral smear showed anisopoikilocytosis, tear-drop cells, and polychromasia, suggestive of hemolysis. The reticulocyte count was elevated (9.6%).

*Biochemistry*: Total bilirubin was 3.1 mg/dL, with an indirect fraction of 2.6 mg/dL. Serum ferritin was low (12.4 ng/mL), suggesting concomitant iron deficiency.

*Imaging*: Abdominal ultrasonography confirmed splenomegaly with normal liver echotexture.

The patient was advised advanced diagnostic tests; however, due to financial constraints and lack of consent from the patient's caregivers, quantitative porphyrin analysis and *UROS* gene sequencing could not be performed. However, the presence of the pathognomonic triad of severe photosensitivity, erythrodontia, and porphyrinuria established the clinical diagnosis of congenital Erythropoietic Porphyria.

## Management

The parents were counseled regarding the chronic nature of the disease and its autosomal recessive inheritance pattern. Recurrence risk in future pregnancies was explained, and genetic counseling was provided. Strict photoprotection was advised, including avoidance of direct sunlight and the use of protective clothing.

The child was referred to the pediatric hematology team, who advised supportive management with monitoring of hemolysis. Chronic transfusion therapy was discussed but deferred, as the patient was clinically stable and not transfusion-dependent at present. The option of hematopoietic stem cell transplantation as a potential therapeutic modality was discussed, with counseling regarding variable outcomes and possible recurrence.

The parents were also counseled regarding government support under the National Policy for Rare Diseases; however, they were not willing to pursue these options. Due to the limited availability of patient support groups in the region, no formal patient advocacy resources could be offered, and the parents were counseled regarding home-based care and follow-up.

## Discussion

Congenital erythropoietic porphyria is a very severe and mutilating form of porphyria. It is an ultra-rare disorder worldwide. In India, the literature is limited to sporadic case reports and small case series, with no established prevalence data, suggesting that the condition remains underrecognized and underdiagnosed in this setting.

CEP is inherited predominantly in an autosomal recessive manner and may occur even in children born to nonconsanguineous parents, as both parents may harbor clinically silent pathogenic variants as heterozygous carriers. Therefore, the absence of consanguinity should not diminish clinical suspicion for CEP in the presence of characteristic features. Although rare de novo mutations have been described, most reported cases result from inherited mutations, underscoring the importance of genetic counseling and recurrence risk assessment in affected families.^1^^,^^4^

At the molecular level, the disease results from the accumulation of water-soluble uroporphyrin I in the skin, which absorbs excitation energy from visible light within the Soret band (400-410 nm), leading to the generation of reactive oxygen species, complement activation, and subsequent tissue damage.^4^

The clinical hallmark of CEP is photosensitivity with blistering over sun-exposed skin, typically presenting in early infancy, although delayed onset may occur. Recurrent blistering predisposes to secondary infection, scarring, and progressive photomutilation of acral structures, including resorption of nasal cartilage and loss of digits, although our patient presented early before such deformities developed. Hypertrichosis, as observed in this case, is a common compensatory response to chronic cutaneous fragility.^2^

Erythrodontia is a pathognomonic feature of CEP. Porphyrins are deposited within the calcium phosphate matrix of dentin and enamel, resulting in reddish-brown discoloration of teeth that fluoresce under ultraviolet light.^5^ The red discoloration of urine observed in CEP is due to massive urinary excretion of uroporphyrin I.

Hematological manifestations result from porphyrin accumulation within erythroblasts, leading to ineffective erythropoiesis and chronic hemolysis. Splenomegaly is a common consequence of ongoing extravascular hemolysis. The severe anemia observed in our patient was likely multifactorial, resulting from hemolysis compounded by nutritional iron deficiency.

The clinical course of CEP demonstrates considerable heterogeneity, with prognosis varying according to disease severity and access to appropriate long-term care. Severe forms presenting in infancy may show progressive disease with major long-term complications, including recurrent blistering, increasing scarring, photomutilation, chronic hemolysis, hypersplenism, ocular involvement, and deformities of sun-exposed structures, potentially resulting in significant functional impairment. Prognosis is influenced by the degree of hematologic involvement, adequacy of photoprotection, transfusion requirements, and access to definitive therapies such as hematopoietic stem cell transplantation, with milder hematologic disease and early preventive measures generally associated with slower progression and improved outcomes. Historically, severe disease has been associated with reduced survival; however, advances in supportive management and therapeutic interventions have substantially improved long-term outcomes, with some patients now surviving well into adulthood.^2^^,^^4^

Diagnosis is ideally confirmed by demonstration of elevated uroporphyrin I and coproporphyrin I levels in urine and plasma, along with markedly elevated erythrocyte porphyrins. Molecular confirmation by UROS gene analysis remains the gold standard.^1^ However, in resource-limited settings, the combination of characteristic clinical features and Wood's lamp fluorescence is highly specific and diagnostically valuable.

The differential diagnosis of infantile photosensitivity and blistering disorders includes hepatoerythropoietic porphyria (HEP), porphyria cutanea tarda (PCT), erythropoietic protoporphyria (EPP), epidermolysis bullosa (EB), xeroderma pigmentosum (XP), and hydroa vacciniforme (Table I). CEP can be distinguished clinically by the characteristic combination of early-onset severe photosensitivity, erythrodontia, porphyrinuria, and fluorescence findings on Wood's lamp examination.^4^^,^^5^

**Table I:** Comparative clinical and genetic features of congenital erythropoietic porphyria and its differential diagnoses^4^^,^^5^

Differential diagnosis	Inheritance pattern	Gene/enzyme defect	Major clinical features	Distinguishing features from CEP
Congenital erythropoietic porphyria (CEP)	Autosomal recessive	*UROS*—uroporphyrinogen III synthase deficiency	Severe photosensitivity, blistering, scarring, hypertrichosis, erythrodontia, porphyrinuria, hemolytic anemia	–
Hepatoerythropoietic porphyria (HEP)	Autosomal recessive	*UROD*—uroporphyrinogen decarboxylase deficiency	Photosensitivity, blistering, scarring, hypertrichosis, variable erythrodontia	Typically less prominent erythrodontia and porphyrinuria; biochemical profile differs
Porphyria cutanea tarda (PCT)	Sporadic or autosomal dominant	*UROD*—uroporphyrinogen decarboxylase deficiency	Skin fragility, vesicles, bullae, hyperpigmentation, hypertrichosis, tea-colored/reddish urine	Usually presents later in life; significant hemolysis and classic erythrodontia are not characteristic
Erythropoietic protoporphyria (EPP)	Autosomal recessive/complex inheritance	*FECH*—ferrochelatase deficiency	Photosensitivity with burning, erythema, edema	Blistering less prominent; erythrodontia and porphyrinuria are not characteristic
Epidermolysis bullosa (EB)	Variable	*KRT5, KRT14, COL7A1*, and related structural protein defects	Recurrent blistering and scarring	Predominantly trauma-induced blistering; lacks porphyrin-related findings
Xeroderma pigmentosum (XP)	Autosomal recessive	DNA repair gene defects	Photosensitivity, pigmentary changes, early cutaneous malignancies	Predominant pigmentary abnormalities without characteristic porphyrin-related findings
Hydroa vacciniforme	Usually sporadic	No specific genetic defect established	Recurrent vesiculopapular lesions on sun-exposed areas healing with varioliform scars	Absence of hemolysis and porphyrin-related findings

Management of CEP is largely supportive, with rigorous photoprotection remaining the cornerstone of therapy. Oral activated charcoal and hypertransfusion regimens aimed at suppressing endogenous erythropoiesis have been used with variable success. Chronic transfusion therapy may be considered in selected cases to suppress erythropoiesis; however, it was not initiated in our patient due to clinical stability. Allogeneic hematopoietic stem cell transplantation has been explored as a therapeutic option, although outcomes remain variable and it is not uniformly curative.

## Conclusion

This case underscores the importance of clinical acumen and the utility of simple bedside tools such as Wood's lamp examination in diagnosing rare genodermatoses. Early diagnosis of congenital erythropoietic porphyria allows timely institution of strict photoprotection and early treatment, which is crucial in preventing severe scarring and photomutilation associated with the natural course of the disease.

## Conflicts of interest

None disclosed.
